# On the possible role of robustness in the evolution of infectious diseases

**DOI:** 10.1063/1.3455189

**Published:** 2010-06-30

**Authors:** C. Brandon Ogbunugafor, James B. Pease, Paul E. Turner

**Affiliations:** Department of Ecology and Evolutionary Biology, Yale University, New Haven, Connecticut 06520, USA

## Abstract

Robustness describes the capacity for a biological system to remain canalized despite perturbation. Genetic robustness affords maintenance of phenotype despite mutational input, necessarily involving the role of epistasis. Environmental robustness is phenotypic constancy in the face of environmental variation, where epistasis may be uninvolved. Here we discuss genetic and environmental robustness, from the standpoint of infectious disease evolution, and suggest that robustness may be a unifying principle for understanding how different disease agents evolve. We focus especially on viruses with RNA genomes due to their importance in the evolution of emerging diseases and as model systems to test robustness theory. We present new data on adaptive constraints for a model RNA virus challenged to evolve in response to UV radiation. We also draw attention to other infectious disease systems where robustness theory may prove useful for bridging evolutionary biology and biomedicine, especially the evolution of antibiotic resistance in bacteria, immune evasion by influenza, and malaria parasite infections.

Unifying principles in biology are rare and challenging to uncover, as they are charged with explaining phenomena across different areas of the biosphere, on different scales. Robustness is a modern concept in biology with the potential to serve as a unifying principle, as it has already been wielded in vastly different contexts, including yeast metabolism, embryology, cancer biology, and many others. In general, robustness describes the capacity for an organism to persist in the presence of perturbations of various kinds. Robustness exists in several forms, with genetic robustness the most provocative among them, describing the ability of organisms to resist phenotypic change in the presence of genetic variation itself, influencing the ability for natural selection to act on heritable genetic information (evolvability). Several recent studies have fortified the importance of testing robustness empirically, where one can detect evolvable differences using various methods. These studies, however, highlight both the opportunities and obstacles involved with the empirical study of robustness. Because many of these studies have utilized microorganisms, the infectious disease paradigm is a candidate area for further application of robustness theory. One can argue that recent findings in several infectious disease systems, including bacterial drug resistance, influenza, HIV, and malaria, are germane to the robustness concept. The hope is that further application of robustness theory might aid in how we study, and treat, infectious diseases of many types.

## I. INTRODUCTION

In biology, robustness describes the relative capacity for a biological system to maintain constancy of phenotype (e.g., population growth, individual development), despite perturbation by mutation (genetic robustness) or by environmental change (environmental robustness).^1–4^ Epistasis is implicit in genetic robustness; a robust genome tends to retain phenotype when a mutation is introduced, whereas the identical mutation is expected to typically alter phenotype when placed in a brittle (nonrobust) genetic background. Both types of robustness are central to evolutionary biology because robustness dictates how organisms respond to environmental challenges, the very crux of natural selection.

**FIG. 1.**
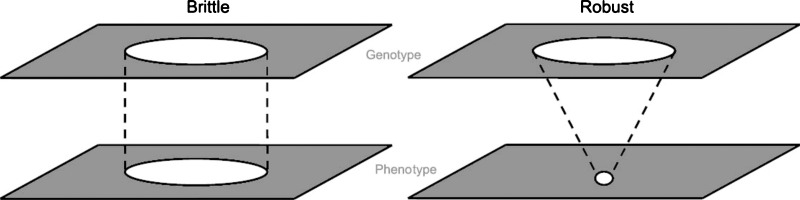
Genotype and phenotype spaces are represented schematically in two dimensions. A brittle organism produces a phenotype that is a reflection of the underlying genotype, whereas a robust organism produces a constant phenotype regardless of the underlying genotype. [Reprinted with permission from M. Félix and A. Wagner, Heredity **100**, 132 (2008). Copyright ©2008 Nature Publishing Group.]

Advancements in the understanding of robustness and its evolution have often arrived through theoretical studies,^5–8^ but empirical studies have made recent in-roads. Experiments using artificial life (“digital organisms,” self-replicating computer programs that can evolve) valuably demonstrated that elevated mutation rates can select for evolved increases in genetic robustness to tolerate mutation, even at the expense of reduced reproductive fitness.^9^ The explanation was that high mutation rates could selectively favor genetic variants that were not necessarily productive and resided on flat regions of the “fitness landscape;” these robust genotypes formed an epistatic network that produced equally fit phenotypes despite mutation-induced movement across the landscape (Fig. 1).^6,8,10^ Other landmark studies have successfully examined robustness by considering phenotypic effects of mutations underlying proteins using computational and *in vitro* approaches.^11,12^

Viruses with RNA genomes are natural systems that typically experience high mutation rates owing to their lack of error-repair during replication. Thus, RNA viruses have proved to be useful and tractable models for studying robustness evolution in biological populations. This work has focused on the success of robust versus nonrobust RNA virus variants when mutation rates are further elevated through exposure to ultraviolet (UV) light and other mutagens,^13^ and on evolved changes in robustness under frequent virus coinfection, which allows buffering of mutational effects via complementation.^14^ Below we review some of the evidence from these studies, and present new findings from an increasingly popular model system for examining evolution of robustness: the segmented RNA bacteriophage ϕ6.^14–16^ In reviewing these results, we hope to highlight the importance of empirical work in RNA viruses for testing theory pertaining to robustness, as well as for better understanding the evolutionary biology and evolvability of infectious organisms in general.

Our second main goal is to review the evidence from biomedically important infectious diseases of humans, describing how the success of various disease agents may be commonly considered in light of their genetic and/or environmental robustness. The importance of robustness has been discussed in the context of noninfectious diseases such as cancers and metabolic syndromes.^17,18^ Here we focus particularly on infectious disease systems: the evolution of antibiotic resistance in bacteria, influenza virus host shifts, and malaria parasite invasion. A review of these studies seeks to further demonstrate the broad importance of robustness to theories of biological systems, and the evolution of infectious disease.

## II. EMPIRICAL EVIDENCE FOR EVOLUTION OF ROBUSTNESS IN RNA VIRUSES

If a population is well-adapted to its environment, almost all mutations should lead to deviations from optimal performance in the selective habitat. Therefore, populations at equilibrium are expected to experience selection for genetic robustness. But study of this process in experimental populations is problematic for at least two reasons: equilibrium states are difficult to achieve (or definitively prove) in lab-evolved populations, and selection for mutational robustness is “second-order” because the benefit is not experienced until offspring carrying mutations arise.^1^ However, theory and artificial-life data^9^ support the idea that genetic robustness should be strongly favored when populations experience elevated mutation rates, suggesting that RNA viruses would be fruitful systems to explore how genetic robustness evolves. In general, success in these systems has come either from manipulating ecological conditions such that robustness becomes less important and evolves to decrease, or from manipulating competition environments to examine whether elevated mutation rates favor genetically robust populations. In both strategies, the ultimate goal was to manipulate treatments and confirm that certain test populations better maintained a measurable phenotype in spite of mutational change, and thus were relatively robust.

Theory on adaptive genetic robustness under elevated mutation rate generally assumes that phenotype expression results solely from the underlying genotype. However, viruses can employ complementation, a mechanism whereby low-fitness genotypes can use, to their advantage, intracellular proteins made by coinfecting strains of high fitness.^19–21^ Coinfection combined with complementation can therefore provide phenotypic buffering in the event of genomic mutations, similar to other buffering mechanisms such as gene duplication and diploidy that might be positively selected because they facilitate canalization in higher organisms.^22,23^ Complementation can buffer against negative fitness effects of deleterious alleles in coinfecting populations of viruses.^20^ This buffering effect introduces an ecologically determined community-level robustness which renders their individual-level robustness less necessary. This logic infers that the degree of coinfection—high multiplicity of infection (MOI; ratio of viruses to cells) versus low MOI—should influence evolution of robustness in virus populations.^14^ We note that many other phenomena consequential for virus evolution can occur as a result of coinfection, especially genetic exchange (recombination) between viruses and selection for virus “cheaters” such as defective-interfering particles.^24^ For brevity we limit our discussion to complementation that occurs during coinfection and its potential role in the evolution of virus robustness.

The segmented RNA phage φ6 is typically grown in the laboratory on the plant pathogenic bacteria *Pseudomonas syringae* pathovar *phaseolicola*. To examine whether high-MOI populations of phage φ6 will evolve decreased genetic robustness against mutations, virus populations were first propagated for 300 generations under high versus low MOI.^25,14^ The expectation was that high-MOI evolved viruses would become more brittle, in comparison to their low-MOI evolved counterparts. To reveal this assumed outcome, clones were randomly chosen from the treatment populations and used to initiate lineages subjected to mutation accumulation via passage through severe bottlenecks (population size=1 individual); this method allows genetic drift to overwhelm selection, such that virus lineages amass mutations that are on average deleterious.^26^ The mean magnitude and variance in fitness changes that occurred because of bottlenecking were statistically compared between lineages founded by presumed robust (low-MOI) and brittle (high-MOI) viruses. Results from the mutation accumulation analysis supported the hypothesis that viruses historically evolved under frequent coinfection became relatively less genetically robust than those evolved under rare coinfection.^14^ One alternative explanation for the observations would be a higher mutation rate in the viruses evolved at high MOI, causing them to experience a greater number of fixed mutations (and hence, greater fitness loss) during the mutation accumulation; however, no evidence suggests that these viruses mutate at rates higher than their counterparts evolved at low MOI.^14,15^ Thus, the study provided the first evidence that genetic robustness could evolve to change in biological populations.

Other experimental studies have used RNA viruses or viroids (RNA viruslike plant pathogens that lack protein-coding genes) as models to study whether elevated mutation rates favor robust genotypes. One study examined two species of viroids that differed in robustness.^13^ The robust viroid species had a low reproductive capacity in plants but a large neutral neighborhood of mutations (fraction of one-error mutations that do not change minimum free energy RNA-secondary structure), whereas the brittle viroid had the opposite combination of traits. It was hypothesized that brittle viroids should outgrow robust viroids when coinfecting plants under ordinary conditions, but the robust viroids should be favored in the presence of a mutagen because they better maintained phenotype (proper structure) despite mutational input. As predicted, under standard growth conditions in host plants, results showed that the brittle viroid was selectively favored. In contrast, when infected plants were subjected to irradiation by ultraviolet C (UVC; short wave UV light of wavelength 280–100 nm), this known viroid mutagen caused the robust viroid to be favored in competition, because it better withstood growth despite mutation.^13^ Thus, this empirical outcome in biological populations was consistent with the earlier findings seen in artificial-life studies.^9^

A second study used an RNA virus of eukaryotes and two different mutagens, but found very similar results to those described above.^27^ Vesicular stomatitis virus (VSV) is an ssRNA virus that is vector-transmitted by some insects and causes disease in certain mammal species.^28,29^ Two diverged populations of VSV were characterized for reproductive growth on tissue culture cells, and for within-population variance among clones for this phenotype; this effort revealed that one population was slow-growing but relatively less variable in clone phenotypes (robust) and the other had the opposite characteristics (brittle).^27^ Whereas standard growth conditions allowed the faster replicating brittle virus population to outcompete the slower replicating robust population, exposure to either of two mutagens favored the robust variant. These results echoed the earlier studies where competition under elevated mutation rates was observed to favor robust populations, even though robustness was associated with reduced reproduction.^9,13^

While several of these studies have provided evidence for extant, evolvable differences in robustness, the literature is generally lacking in examples of direct selection for robustness in biological systems. In an effort to examine direct selection for RNA virus resistance to UVC damage, here we present new data from a short-term experimental evolution study with phage φ6. These results underscore some of the biological, methodological, and conceptual barriers to observing direct selection for robustness in RNA virus systems.

**FIG. 2.**
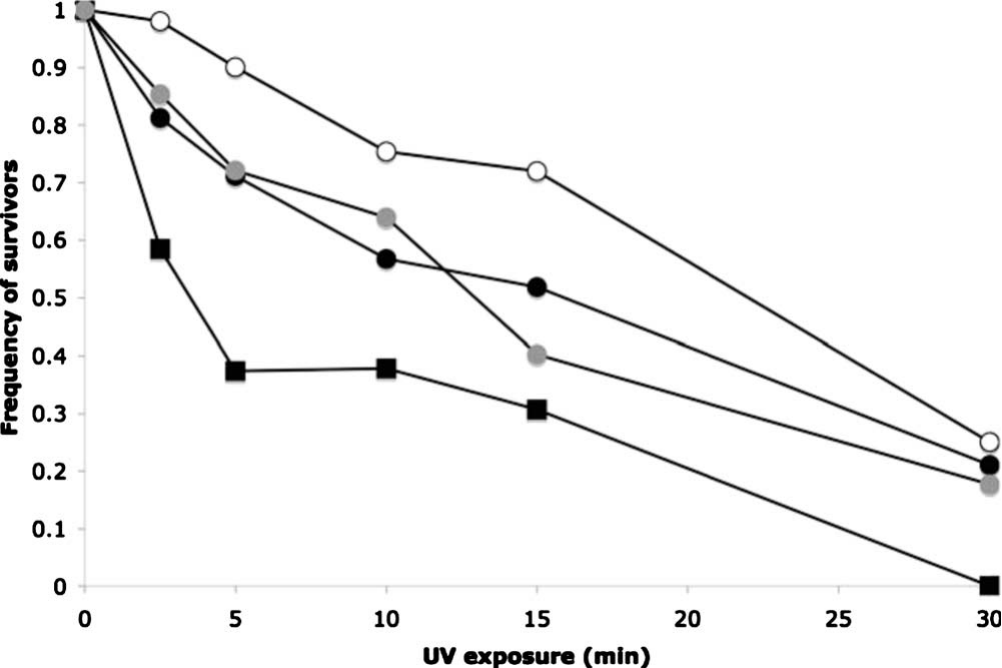
Death curves for virus strains in response to differing dosages of UVC exposure. Wild type RNA phage φ6 (filled circles) better withstands UVC degradation than does DNA phage φX174 (filled squares). A phage φ6 population experimentally evolved for 20 generations under 2.5 min UVC exposure (open circles) survives better than its ancestor, except under prolonged dosage. In contrast, a representative phage φ6 control population (gray circles) evolved in absence of UVC exposure presents a death curve nearly identical to the ancestor. See text for details.

UVC is well-known to impact RNA viruses negatively^30,31^ and previous studies have directly examined the mutagenic effects of UV radiation on RNA genomes.^13,32^ Furthermore, evidence from the biochemical and biophysical literature suggests that UV radiation can interfere with RNA replication fidelity.^33–35^ Regardless, preliminary experiments showed that UVC exposure for periods up to 30 min greatly increased mortality in wild type phage φ6 (Fig. 2), indicating that this environmental effect should produce strong selection for UVC resistance in populations of the virus. We note that identical UVC exposure was even more damaging in another well-studied virus, the ssDNA phage φX174 (Fig. 2); this difference suggested that RNA phage φ6 may be relatively more robust to effects of UVC than other viruses, echoing our earlier suggestion that the virus was fairly robust to mutation accumulation.^14^ For this reason, experimental evolution of phage φ6 under UVC exposure may provide a conservative test for whether virus populations can adapt to resist the debilitating effects of UV.

A single clone of wild type phage ϕ6 was used to found three replicate populations in each of three experimental treatments (nine populations total), where UVC exposure was manipulated using our published methods.^16^ A 200 ml aliquot containing $∼10^{6}$ plaque-forming units (pfu; i.e., viable virus particles) of each population was suspended in Luria broth culture medium in the absence of cells. Aliquots were then placed in wells of a flat-bottomed polystyrene 96-well plate. A UV illuminator (Spectroline Long Life Filter) was placed over the 96-well plate to expose the virus samples to UVC emission. “High dosage” treatment exposed populations to UVC for 15 min, “low dosage” treatment exposed them for 2.5 min, and the “control” treatment populations were never exposed to UVC. Following this manipulation, each population was mixed (1:1 volumetric) with *P. phaseolicola* bacteria (i.e., $∼4\times 10^{9}$ cells) and incubated at 25 °C for an additional 120 min, sufficient time for at least one virus generation consisting of cell attachment/entry, intracellular replication of virus progeny, and cell lysis (death) that bursts the cell to liberate offspring. A typical burst in phage φ6 produces ∼200 viral progeny per infected cell^36^ (we note that the host bacteria were never exposed to UVC, eliminating the possibility that UVC negatively impacted cell physiology or caused host mutagenesis). After 120 min, the mixture was centrifuged to pellet cells, and the supernatant was filtered to harvest a cell-free virus lysate containing up to $∼2\times 10^{8}\;\text{pfu}$ of virus progeny. The lysate was then stored at 4 °C for 22 h. The lysate was diluted 100-fold and used to initiate the next passage where the phage population was again subjected to treatment or control conditions, and allowed to infect naive bacteria freshly cultured from frozen stock (i.e., to eliminate possibility of phage-bacteria coevolution). Thus, experimental populations were challenged to produce sufficient progeny to sustain themselves in the face of the daily 100-fold dilution; UVC was an environmental stressor that could reduce the likelihood of this sustainability unless viruses responded through adaptation. A total of 20 passages (i.e., 20 generations) occurred in the short-term selection experiment, and samples at each passage were stored at −80 °C for future analysis.

At the end of the study, replicated (n=4) assays were performed to gauge whether the survival of treatment populations had changed during the experiment. Evolved populations were measured for frequency of surviving virions, in assays where samples containing $∼10^{6}\;\text{pfu}$ of virus were exposed to UVC for six different time periods: 1, 2.5, 5, 10, 15, and 30 min. Results showed that after 20 passages the death curves for the populations in the control treatment were highly similar to that presented by the wild type phage φ6 ancestor; Fig. 2 shows a representative outcome for one of the control populations. In contrast, we observed that all three lineages in the high dosage (15 min UVC exposure) treatment went extinct by passage 10 of the experiment (data not shown). These data were informative because they indicated that the strong selection created through high dosage of UVC exposure was too overwhelming for viruses to be ecologically sustained, and/or to experience spontaneous genetic variation useful for meeting the environmental challenge. The latter outcome clearly demonstrated an evolutionary constraint for the virus. Last, all of the evolved populations in the low dosage (2.5 min UVC exposure) treatment were extant by end of the study. However, only one of these three populations exhibited a death curve that differed from the ancestor, as shown in Fig. 2. This result proved that a response to UVC selection was possible in phage φ6 under our low dosage conditions. Furthermore, the data interestingly showed that the adaptation caused ∼17% gain in survival (on average) relative to the ancestor at dosages away from the selection challenge of 2.5 min (i.e., 1, 5, 10, and 15 min). However, this response on the part of the one lineage was not overly impressive, because the same 50% mortality as the ancestor was evident under 30 min UVC exposure, demonstrating an additional constraint in the virus even though an adaptive response occurred at low dosage (Fig. 2).

Given the overall modest response in phage φ6 populations subjected to strong selection for changes in the UVC survival phenotype, the available evidence strongly suggests that pre-existing constraints prevent the virus from easily adapting. Thus, we did not observe adaptive increases in virus resistance to UV degradation, presumably involving mutagenesis. However, the experimental evolution was of relatively short duration, and additional selection might produce adaptive resistance to UVC damage. Nevertheless, a lack of adaptive response may be unsurprising in phage φ6, because elsewhere we showed that genetically robust and brittle strains of the virus generated in the aforementioned MOI experiment did not statistically differ when assayed for UVC survival.^16^ Thus, neither direct selection (current findings) nor indirect selection for robustness against UVC damage was evident in this study system. These observations may be explained by a relatively high level of robustness to UV damage in phage φ6, which has already run the gamut of selection, creating a constraint for the virus to improve any further. One ecological explanation may be that the virus naturally infects plant pathogenic bacteria that colonize leaf surfaces,^37^ suggesting that prior adaptation to resist UV damage might have precluded observations of further adaptive response in the laboratory.

This study reveals the potential pitfalls to overinterpreting empirical results in the attempt to study robustness. First, claiming that an organism has directly evolved robustness through natural selection requires a nuanced understanding of how a given environmental stressor impacts an evolvable entity. Such knowledge can be dubious in the RNA virus context, as stressors such as UVC might impact RNA viruses in multiple ways.^30–32^ Even further, if a test population evolves an adaptive response to an environmental challenge, one has to be cautious in distinguishing resistance from robustness. While both involve the ability to withstand the influence of perturbations, robustness describes a more generalized response that affects performance in multiple environments^1,2^ and evolvability.^15,16^

## III. TESTING RELATIONSHIPS BETWEEN ROBUSTNESS AND PATHOGEN EVOLVABILITY

The existing empirical evidence for evolved changes and selective advantages in robustness reinforces the importance of microbial systems in the study of evolutionary biology. But later work examining the relationship between robustness and evolvability is perhaps even more relevant for elucidating how infectious diseases evolve. On the one hand, the ability for robustness to allow phenotypic constancy in the face of environmental and mutational perturbation provides obvious benefits, such as reliable cellular function, individual development, and offspring reproduction. However, rigidity in the face of change may pose problems; because natural selection acts on phenotypic variation, robustness that buffers this variation could impede evolution by natural selection. These conflicting necessities force organisms to strike a balance between robustness and evolvability, the capacity to adapt. By examining this balancing act, we may learn whether evolvability can itself evolve; i.e., whether natural selection can exert a second-degree effect on evolution. Below we briefly describe two recent experiments on the relationship between robustness and evolvability.

One experiment showed that genetic robustness improved evolvability of phage φ6 populations when heat shock was used as an adaptive challenge.^15^ Phage ϕ6 is typically cultured at 25 °C, and exposure to 45 °C heat shock for as little as 5 min leads to ∼20% survival (%S) in populations of the virus. Robust and brittle clones of the virus from the aforementioned MOI experiment^14^ were used to found lineages passaged for 50 generations of experimental evolution with periodic (every fifth generation) exposure to 45 °C heat shock. At the end of the study, we measured mean %S at 45 °C measured for each founding clone and its derived end point population to estimate Δ%S, the change in percent survival after heat-shock selection. The results showed that the lineages founded by robust genotypes were more evolvable (greater Δ%S), indicating that robustness promoted evolvability in phage ϕ6, under the test conditions. The explanation was that robust genotypes of phage ϕ6 may have proteins that better tolerate mutations while maintaining proper folding especially at moderate temperatures,^16^ similar to suggestions from *in vitro* studies where less-sensitive (relatively robust) proteins seem more likely to maintain their function in a new environment where innovation is needed.^11^ That is, despite equivalent sensitivity to 45 °C heat shock in the robust and brittle founding strains, the robust viruses may have proteins that tend to be capable of undergoing mutations while maintaining their proper folding when 45 °C constitutes a selective environment.^15^ Whether this exact relationship extends to other novel environments has yet to be explored. But the fact that a positive relationship exists at all is essential for tests of existing theory,^8^ and begs the question of whether the observed relationship between genetic robustness and evolvability extends to evolution of human pathogens, as implicated in recent claims of evolved increases in genetic robustness in HIV.^38^ Antiviral therapy consisting of chemical mutagenesis that induces an insurmountable mutational load seems promising for treatment of RNA virus infections.^39–41^ But such measures may select for RNA viruses to evolve altered polymerases with improved replication fidelity that reduces genomic mutation rates.^42^ Alternatively, viruses may be selected to evolve mechanisms of genetic robustness where high mutation rates are preserved but phenotypic effects of the mutagen are buffered. This outcome is perhaps unlikely given the empirical data thus far.^43^ However, further data involving a variety of different viruses are warranted, and the results from experimental evolution studies caution that robustness may be positively related to evolvability and to competitive superiority in RNA viruses.^15^

A second experiment centered closely on the potential link between robustness and pathogen evolvability on novel hosts. Environmental robustness of a virus may be defined in terms of its host generalization (host range, the number of host types it can productively infect).^44^ Theory predicts that evolved generalization may be particularly useful if organisms tend to encounter environments that change unexpectedly.^45^ That is, generalists may be favored by selection because they are better predisposed to survive and give rise to successful progeny in the face of variable environments, relative to specialists that possess a narrow niche. This idea may explain why pathogens with a pre-existing broad host range seem better able to emerge on novel hosts, relative to specialized pathogens.^46–48^ To test this idea directly, we used populations of VSV that evolved differences in host use because of prior selection in constant versus variable host environments.^49,50^ The formal prediction was that VSV populations experiencing direct selection for host range would have higher mean growth and less variance in mean growth on a collection of challenge hosts, compared to VSV populations that were either relatively specialized or indirectly selected for host range.^51^ When challenged to grow on four novel hosts *in vitro*, the viruses that had been selected for generalism exhibited higher or equivalent host growth, lower among-population variance in host growth, and lower variance in population growth across hosts. Thus, these three predictions relating to the hypothesis were generally supported because direct selection for host breadth more often allowed successful emergence. The results suggested that determination of current niche breadth should be further investigated as a potentially useful indicator in predicting pathogen emergence.^51,52^

Because these many recent empirical breakthroughs in the study of robustness have involved microbes, one might predict that infectious disease is the primary biomedical realm where these robustness results might have an immediate, practical impact. Below we describe three examples of biomedically important human disease systems where robustness theory appears highly relevant.

## IV. ROBUSTNESS AND THE EVOLUTION OF ANTIBIOTIC RESISTANT BACTERIA

The effects of evolution are sometimes difficult to perceive in daily life, making public skepticism of the existence of evolution all the more challenging to address. Perhaps the foremost example used to counter these notions is the widespread problem of resurgent diseases that had been previously treated through antibiotics, especially evolved resistance in bacterial pathogens. Although naturally produced antibiotics evolved in microbial communities long before humans appeared as a species, the medical realm began earnestly administering antibiotic drugs only since their discovery in the 1950s. Widespread production and utilization of antibiotics in medicine and agriculture, however, has created a massive uncontrolled experiment in which bacteria have been selected to harbor antibiotic resistance genes, making existing therapies largely ineffective in many clinical circumstances.

Bacterial resistance to antibiotics and other harmful substances (e.g., heavy metals) often occurs through genes carried on plasmids: autonomously replicating DNA elements that are nearly ubiquitous in bacterial populations. Individual plasmids can sometimes include multiple genes conferring resistance to a variety of antibiotics. Also, plasmids can effectively spread within and among bacteria populations and species through conjugation (cell-to-cell horizontal transfer). Together, these features create the capacity for rapid spread of multiple drug resistance in bacteria. Furthermore, plasmid-borne mechanisms (e.g., postsegregational killing) often exist to ensure that vertical transfer (plasmid inheritance) is reliably maintained across host-cell generations. For these reasons, plasmid biology has undoubtedly contributed to the current crisis of ineffective antibiotic therapy.

Plasmid-bearing bacteria that are resistant to a wide variety of antibiotics can be defined as relatively environmentally robust, compared to bacteria strains unable to grow in the presence of antibiotic challenges. Whether this difference arose largely through selection acting at the level of bacteria, or purely at the level of plasmids, is unclear. In the former case, acquisition and maintenance of a large number of resistance genes could be evolved “bet-hedging” in bacteria, where selected retention of these alleles occurs despite only rare circumstances when they are actually needed to protect against antibiotics. Alternatively, selection may be acting purely at the level of plasmids, favoring infectious genetic elements that collect resistance genes which may be beneficial for “paying the rent” to their bacterial hosts, as plasmids cannot easily control the host backgrounds and environments in which they reside. Last, selection may be acting on the symbiotic microbe created through plasmid/cell association, where the combined interests of plasmids and their hosts may sometimes coincide and other times conflict. Regardless, environmentally robust bacterial pathogens of humans must have been selectively favored during the past half-century, or the ineffectiveness of antibiotic therapy would not be a prominent issue today.

The evolutionary consequences of environmental robustness in resistant bacteria have not been widely addressed. Bacteria able to robustly grow in a variety of antibiotic environments are generalists according to the ecological definition of niche breadth. This niche breadth may have been directly molded through selection if the bacteria descended from a lineage exposed to the various antibiotics. Alternatively, the niche breadth may be fortuitous if the bacteria obtained a plasmid harboring multiple-resistance genes that ran the gauntlet of selection in other contexts. No matter the circumstance, we noted above that some theory predicts evolved generalization may be particularly useful if organisms tend to encounter highly variable environments.^41^ The phenomenon of drug cross-resistance and possible evolutionary modification of a resistance allele allowing survival in altered circumstances (i.e., evolutionary innovation) lend plausibility to the concept that multiple-resistance strains by an evolvability advantage would better thrive in the face of novel drugs. We are unaware of any studies addressing the relationship between number or diversity of resistance genes in bacteria and the evolvability of bacterial pathogens in new environments. Such research would be highly useful for examining the evolutionary processes underlying the widespread success of resistant bacteria.

We note that existing data already suggest that genetic robustness is relevant to the evolution of antibiotic resistance in bacteria. Research shows that compensatory mutation is an important underlying genetic mechanism for maintenance of resistance in the absence of antibiotic selection.^53^ This epistasis mechanism explains how a resistance gene that is costly for bacterial growth in absence of an antibiotic may be compensated by one or more mutations in the plasmid or bacterial chromosome, effectively eliminating the cost of carrying the resistance gene (or the plasmid on which it resides) when no antibiotic is present. If a bacterial strain contains a compensatory mutation that interacts to reduce the cost of various different resistance genes, then this epistatic mutation would provide an example of a genetic-robustness allele responsible for environmental robustness across antibiotic environments. For example, the multiple antibiotic resistance locus in *E. coli* and several species of *Salmonella* is involved with production of an efflux pump that removes various antibiotics from the cell, affording low-level clinical resistance.^54^ However, this mode of resistance can be rather costly if transport in and out of the cell is not tightly regulated. Evidence suggests that the low-level resistance may be a stepping-stone to more finely tuned evolved mechanisms allowing greater resistance,^55^ and we can conceive that genetic changes simultaneously compensating to reduce the cost while maintaining the broad resistance would provide an example of a genetic-robustness allele.

## V. ROBUSTNESS AND MODERN RNA VIRUS PANDEMICS

Many of the above studies suggest ways that robustness might play a role in disease pathogenesis and treatment efficacy, and how robustness might be linked to emergence of future viral diseases in humans. However, contemporary examples already indicate that robustness theory is helpful in elucidating evolutionary dynamics of important RNA virus diseases. One example is the occurrence of flu pandemics in humans, a substantial public health concern in the last century.

**FIG. 3.**
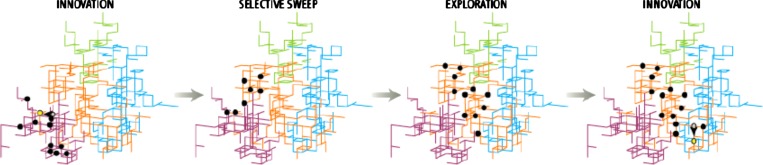
Robustness in gene networks of influenza A HA genes. Phenotypes are genetically robust, allowing genotypes to drift through genotypic neutral space before instant shifts in phenotype. Such a scenario is a demonstration, from the actual epidemiological literature, of how genetic robustness can facilitate evolvability in infectious disease agents. [From E. van Nimwegen, Science **314**, 1884, 2006. Reprinted with permission from AAAS.]

Recent studies suggest that strains of influenza A virus can spread through human populations because they form a neutral network of virus genotypes that share the same phenotype.^55,56^ Essentially, this network allows the virus to drift through neutral space connected via mutation, until a single mutation shifts to a different serotype (Fig. 3). This shift then facilitates escape from host immunity, and fosters the ability for the virus to infect a new class of susceptible hosts. Although these studies make no explicit mention of the role of such genetic robustness in flu epidemics,^55,56^ the available evidence strongly suggests that the phenomenon is relevant in this case of influenza strain H3N2. Genetic robustness in the hemagglutinin (HA) gene of strain H3N2 allows the virus to drift through neutral genotypic space without a change in phenotype until it reaches a rapid saltational phenotypic shift that permits the ability for strains derived from this virus to reemerge in humans. This model is compatible with other robustness-evolvability links outlined in the literature.^57,7,8^

Judging from phylogenetic and molecular evolution analyses of clinical isolates, it appears that the HA gene of influenza A virus appears to be under strong selection by the human immune system,^58^ and perhaps this has been true for much of the evolutionary history of flu disease in humans. The genetic robustness of influenza viruses and the accompanying structure of neutral genetic networks probably arose to foster the epidemiological spread of the virus in humans and/or other animal hosts. The size of these neutral networks is of consequence in the evolution of influenza, as suggested by modern robustness theory.^8^ A HA gene with relatively low robustness would feature a small neutral network; this characteristic might cause the virus to molecularly evolve rather quickly, perhaps outpacing the availability of susceptible individuals in a host population, slowing or halting spread of the virus. But a relatively larger neutral network would afford greater robustness of HA genes in influenza, affording long-term persistence of the virus as it drifts neutrally through sequence space before fortuitously achieving a genotype with a beneficial novel surface antigen that promotes reemergence.

Genetic robustness in the genetic networks of influenza A virus might have an environmental robustness correlate. Influenza A virus is notorious for its characteristically broad host range, including various bird species and mammals such as domesticated pigs whose proximity to humans may spur pandemics.^59–61^ As noted earlier, relatively broad host range of a pathogen may be defined as environmental robustness. The ability for influenza A viruses to traverse the aforementioned neutral network of genetic robustness may have an important environmental robustness component. The virus may be able to easily create a robust neutral network of genotypes associated with attachment/entry of various host cells due to intrinsic environmental robustness that evolved via selection to infect a variety of host environments. In influenza A virus, whether environmental robustness preceded genetic robustness, or vice versa, is difficult to know. But environmental robustness of viruses such as influenza A virus undoubtedly fosters the ability to reside in various host species that may serve as reservoirs that allow occasional spillover into a species of interest such as humans.^52^

Robustness may also be relevant in explaining aspects of the current AIDS pandemic. A global increase in robustness has been invoked to explain an apparent global decline in the virulence of HIV-1 strains.^38^ Although it was not discussed extensively, the robustness concept in this example seems to be environmentally, not mutationally, determined. The argument is that a perceived decline in HIV-1 virulence might be due to generally evolved increase in environmental robustness of the virus, owing to its worldwide spread. The virus has thus encountered variable subpopulations of humans and has undergone selection for environmental robustness, which coincides with improved performance across environments at the expense of reduced reproduction (i.e., virulence) on average. This explanation assumes that the stated tradeoff in viral traits is a generalized phenomenon, and that results for increased genetic robustness at the expense of reduced reproduction^9,13^ apply in the case of environmental robustness as well.

## VI. ROBUSTNESS OF THE INVASION MODULE IN MALARIA PARASITES

Regardless of the disease system, pharmacology involves the use of small molecule inhibitors of biomolecular interactions, and we believe that robustness may be a highly relevant concept in the identification of potential drug targets. This logic begins with identifying in pathogen systems the modules that may serve as good targets versus those that would be poor targets for pharmacological intervention. Presumably, poor targets would be modules that are robust to perturbations, pharmacological agents included.

When designing drugs to treat infections, the invasion step is where the pathogens might be most vulnerable to pharmacological intervention. This idea is supported by studies in which parasite exposure to chemical compounds neutralizes binding to host receptors, fortifying the importance of invasion to the establishment of a clinical infection.^62,63^ Thus, many therapeutic measures against a variety of pathogens rely on this vital receptor-ligand interaction. Virus examples include the seasonal influenza vaccine, and fusion inhibitor compounds, a class of anti-HIV therapies.^64,65^

**FIG. 4.**
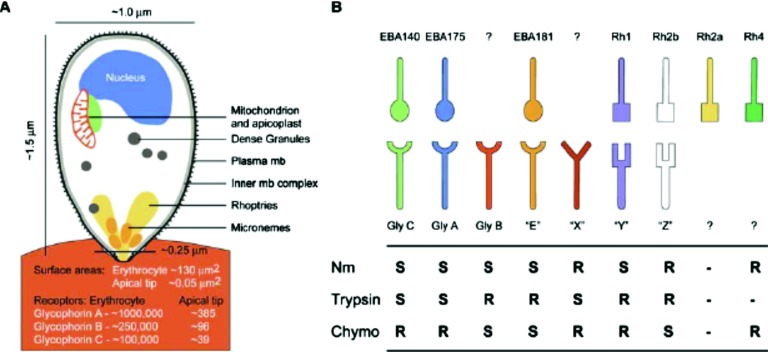
Redundancy in the invasion module of *Plasmodium falciparum*. If a given erythrocyte receptor is inactivated because of the presence of an enzyme, such as neurominidase (“Nm”), trypsin, or chymotrypsin (“Chymo”), there are other pathogen antigens that can bind to other erythrocyte receptors, facilitating invasion. [From J. Baum *et al.*, PLoS Pathog. **1**, e37 (2005).]

In nonvirus parasites the situation is more complicated, because the process of invasion is a complex module involving several different effectors. One possible example of a robust invasion module is in *Plasmodium falciparum*, the causative agent of the most deadly form of human malaria. In *P. falciparum* and the apicomplexan parasites, an apical complex is central to the invasion process, which includes multiple steps involving protein interactions and temporally and spatially regulated expression of proteases and polymerization machinery.^63,66,67^ Given the importance of erythrocytes as cells where parasite replication occurs, one might expect that *P. falciparum* interactions with erythrocytes should be particularly critical in a robustness module for the pathogen. In fact, studies show that the erythrocyte-attachment process involves redundancy in ligands used for binding of the pathogen to erythrocyte receptors.^68,62^ This evolved strategy might have been selected in *P. falciparum* because erythrocyte receptors are highly polymorphic, perhaps among the fastest evolving genes in the human genome.^69,70^ Research shows that *P. falciparum* utilizes a plethora of ligands to bind erythrocyte receptors.^62,68,70^ Interestingly, a “molecular hierarchy” may be the ideal term to describe ligand usage in *P. falciparum*; the pathogen’s invasion module may consist of a hierarchy whereby some ligands used in attachment are preferred over others, with the switch in usage only taking place when a ligand higher up in the hierarchy is ineffective (Fig. 4).^68^ This observation may be usefully couched in terms of robustness, because redundancy is a possible mechanism for constructing a robust biological system. To ensure that an essential function is carried out, a biological system can be organized to contain several alternative pathways in the event that one or more become unavailable or dysfunctional. This evolved strategy decreases the probability that an environmental perturbation disrupts performance.

## VII. CONCLUDING REMARKS

The concept of robustness has the continued potential to shift paradigms in biology by challenging assumed relationships between genotypes and phenotypes. Here we have highlighted some of the empirical evidence for evolution of robustness, and discussed its relevance for evolution of infectious diseases, particularly in humans. Despite the wide-reaching importance of robustness theory in biology and other disciplines, we emphasize that the origins and implications of robustness may be highly context specific. The underlying causes of diseases in humans and other organisms are manifold. To wield the robustness concept effectively in the evolution of infectious diseases, sufficient understanding of how ecological history might have selected for robustness is warranted. Robustness has often resided in the realm of mathematical theory, but future work would benefit from collaborations between empiricists and theoreticians to test explicitly predictions using tractable models, or disease pathogens. These efforts would help to elucidate further the role of robustness in past evolution of parasites such as influenza A virus and *P. falciparum*, and to predict how it might play a role in their future evolutionary change.

Empirical evidence for unifying principles in biology is often sought, but is not easily achieved. One consequence of earth’s biodiversity is that exceptions to general rules are often found if one searches hard enough. For example, a popular notion in biomedicine was that parasites should inevitably evolve to become mutualistic to their hosts, because this would afford long-term survival of a parasite species that might otherwise force its host into extinction. However, theory and experiments later overturned this conventional wisdom by demonstrating that infectious parasites may evolve greater or lesser virulence (damage to the host) depending on their unique ecologies.^71–74^

Similarly, it should be expected that evolution of robustness and its underlying mechanism might differ substantially across the biosphere. Therefore, caution is warranted when attempting to draw widespread conclusions from the handful of valuable empirical studies on evolution of robustness. Perhaps it is possible to construct a universal theory of robustness that applies everywhere, but this is unknown. Our hope is that the exciting studies and biological examples presented here will further motivate researchers in basic science and biomedicine to consider the potential importance of genetic and environmental robustness in a great variety of biological systems. With this impetus, we may amass enough empirical data to help bridge the intellectual gap separating abundant theory on robustness evolution and application of this thinking to applied problems in biomedicine and public health.
